# Recent advances in urologic surgical techniques for pyeloplasty

**DOI:** 10.12688/f1000research.15866.1

**Published:** 2019-03-15

**Authors:** Mikolaj Mendrek, Thomas Alexander Vögeli, Christian Bach

**Affiliations:** 1 Departement of Urology, University Hospital Aachen, Aachen, 52074, Germany

**Keywords:** pyeloplasty, ureteropelvic junction obstruction, surgical techniques, laparoscopy

## Abstract

Pyeloplasty is one of the most common urological reconstructive interventions. Since the presentation of the first open pyeloplasty by Anderson and Hynes in 1949, the management of ureteropelvic junction obstruction has dramatically developed. The most immense progress was made in the 1990s with the introduction of laparoscopy. A multitude of new minimal surgical techniques have been introduced since then. In the last few years, the innovations were based on refinement of already-existing techniques and technology. With this aim, single-port surgery, three-dimensional vision for laparoscopy, robotic technology, and alternative techniques for creating the anastomosis-like fibrin glue have been introduced. This unsystematic review is timely, and the scientific interest is to present and discuss some of the latest advances in surgical techniques and different approaches for the intra- and post-operative management in pyeloplasty. To the best of our knowledge, this is the only review looking at the recent advances in urological surgical techniques for pyeloplasty during the last few years with a focus on new technology and surgical techniques.

## Introduction

The most common reason for congenital hydronephrosis is ureteropelvic junction obstruction. Its prevalence ranges from 1:1500 to 1:500 cases in newborns and affects mostly males (male-to-female ratio of 2:1) ^1, 2^. It occurs in 60% on the left side, but in 10% to 40% both sides can be affected. Based on the etiology, ureteropelvic junction obstruction can be divided into intrinsic and extrinsic. Depending on the etiology, the medical history and the symptoms of the patients can vary widely. In the fetal period, up to 48% of hydronephrosis can be caused by ureteropelvic junction obstruction so that this malformation can often be diagnosed already pre-natally and directly post-natally. On the other hand, there are patients who present first symptoms later in life, even at 60 or 70 years of age. This heterogeneity of patients makes the management of ureteropelvic junction obstruction a challenge for both pediatric and adult urologists. Consequently, a multitude of surgical techniques have been developed ^1, 3^. In this article, we will focus on the most recent advances, especially those aimed at treating adults.

## A brief history of surgical approaches to ureteropelvic junction obstruction

The high prevalence of ureteropelvic junction obstruction makes pyeloplasty one of the most common urological reconstructive interventions. Many techniques have been described since 1886. However, the dismembered pyeloplasty technique elaborated by Anderson and Hynes in 1949 ^4^ has become a gold standard given its reproducible favorable outcomes. A success rate of 90% to 100% has been confirmed in long-term follow-up studies ^5^. Nevertheless, management of ureteropelvic junction obstruction has significantly evolved over the previous century with the advent of minimally invasive surgery ^6^.

As alternatives to the open surgery, minimal access techniques have been developed. They permit clinicians to avoid the drawbacks of the loin incision used to perform a traditional open pyeloplasty: post-operative pain and therefore prolonged reconvalescence and poor cosmetic result. The endourological techniques such as endopyelotomy, endoballoon disruption, or retrograde incision with the Acucise device, however, did not become established, mostly due to the poorer success rate of around 80% and many contraindications and limitations ^7^. The real breakthrough was made in 1993 by Schuessler *et al.* ^8^ with the presentation of a laparoscopic dismembered pyeloplasty. The laparoscopic technique described in adults was the principles known from open surgery analogous to the Anderson–Hynes procedure. This technique became the first minimally invasive intervention for treatment of ureteropelvic junction stricture which was generally accepted, reaching the success rates of the open pyeloplasty in 10 years’ follow-up ^9^. Two years later, in 1995, Peters *et al.* ^10^ published a successful laparoscopic pyeloplasty in a 7-year-old patient, setting a new standard for pediatric urology. Nowadays, laparoscopic procedures are performed even on children less than a year old, and a systemic review and meta-analysis have proven their excellent outcomes ^11, 12^. Nevertheless, the availability of a laparoscopic approach to pyeloplasty is still limited to experienced, high-volume centers, mostly because of the high technical demands on the surgeon, who requires advanced laparoscopic skills ^13^. To facilitate complex laparoscopic procedures, robotic technology was introduced for urology, and the first robot-assisted radical prostatectomy was performed in 2000. Since then, the robotic system has been used for other procedures such as nephrectomy or pyeloplasty ^14^. Magnified three-dimensional (3D) vision, tremor reduction, motion scaling, extended range of motion, and better ergonomics helped to solve the most complicated part of pyeloplasty, namely the intracorporeal anastomosis suture ^15^. According to one of the most important systematic reviews and meta-analyses by Braga *et al.* ^16^, the robotic-assisted and conventional laparoscopic pyeloplasties are equivalent with regards to post-operative urinary leaks, hospital readmissions, success rate, and operative time. At the same time, Wang *et al.* ^17^ showed that robotic-assisted pyeloplasty has a shorter suturing time in comparison with the laparoscopic approach with the equivalent perioperative results.

## Introduction of single-port surgery

The current minimal access techniques of laparoscopy and robotic-assisted surgery are under constant development. Technical advancements in instrumentation brought us to the point of single-port surgery. The invention of single-port surgery came with the desire to minimize surgical trauma, thereby reducing recovery time and post-operative pain, incidence of abdominal adhesions, and incisional hernias. Having fewer access points to the abdominal cavity promises to minimize port-related complications such as bleeding, infections, and abdominal scars and has better cosmetic results ^18, 19^. The term single-port surgery, however, covers many different technical solutions which can potentially be used for pyeloplasty. These are laparo-endoscopic single-site surgery (LESS), natural orifice transluminal endoscopic surgery (NOTES), laparoscopic single-incision triangulated umbilical surgery (SITUS), or even robotic-assisted laparo-endoscopic single-site surgery (R-LESS) ^20^. In 2007, 10 years after the first single-port cholecystectomy ^21^, the first single-port transumbilical nephrectomy and pyeloplasty were described ^22^. For pyeloplasty, the most extensively studied single-port technique is LESS ^23^. Both Stein *et al.* ^24^ and Tugcu *et al.* ^25^ proved that there is no difference in success rate, blood loss, transfusion rates, or hospitalization time between the laparo-endoscopic single-site pyeloplasty and conventional laparoscopic pyeloplasty. However, a higher patient satisfaction, faster recovery, better aesthetic results, and a lower degree of post-operative pain were observed in the LESS group. On the other hand, the median operative time was significantly longer in the LESS pyeloplasty group. At the same time, other authors ^26, 27^ showed similar operating times for LESS compared with conventional laparoscopic pyeloplasty. Naitoh *et al.* ^26^ demonstrated that laparo-endoscopic single-site pyeloplasty can be used for both pediatric and adult patients. The loss of triangulation, clashing of the instruments, and long learning curve are the drawbacks of LESS. To overcome those limitations, the concept of laparoscopic SITUS was introduced ^28^. It combines the common principles of classic laparoscopy with the minimal invasiveness of LESS. Habicher *et al.* ^23^ reported on SITUS pyeloplasties and showed that it could be an attractive alternative to conventional laparoscopy and a viable competitor of single-port surgery.

As the classic LESS technique was found to be highly challenging (even for experienced laparoscopic surgeons), the da Vinci robotic platform was seen as a promising tool to overcome some of these challenges, such as the steep learning curve and difficult intracorporeal suturing ^19, 29^. Unfortunately, the early reports revealed only a marginal benefit of this approach, mostly because of the difficulties of triangulation and the instruments clashing ^30^. This triggered numerous efforts to overcome those obstacles by developing semi-rigid robotic instruments, flexible endoscopes, curved trocars, and multichannel ports ^18^. The surgical approach has also been reconfigured. Olweny *et al.* ^29^ used a GelPOINT access device (Applied Medical, Rancho Santa Margarita, CA, USA) for all robotic trocars with the robotic camera in a 30° upward view. In this configuration, the da Vinci Si surgical robot provided results similar to those of conventional LESS pyeloplasty in terms of post-operative outcomes and complications but with significantly longer mean operative times. With almost the same technique and instruments, another group ^31^ replicated the outcomes, proving safety, feasibility, and quick post-operative recovery time of this reconfigured robotic-assisted pyeloplasty. Another highly interesting technique was presented in a prospective, multicenter study of R-LESS pyeloplasty using a new, robotic, single-site platform with 5-mm flexible instruments. This technique was shown to have a competitive operative time and overall success rate comparable to those of the laparoscopic or open procedure ^19^.

## Advances in laparoscopy: three-dimensional laparoscopic systems

Hand–eye coordination is a challenge in laparoscopy as a 3D reality in observed in 2D vision. To overcome those limitations, 3D visual systems have been developed. The first steps in urological 3D laparoscopy were already made in the early ’90s. However, the 3D laparoscopes and video systems of that time did not improve the vision or surgical performance, facilitate laparoscopic operations, or reduce operating times, mostly because of the poor image quality ^32^. This is the reason why this technology was not implemented in everyday life at that point in time. Recently, more advanced 3D imaging systems have provided a stereoscopic vision which delivers a more realistic depth perception. That revived interest in this technique ^33^. In 2015, Sørensen *et al.* ^34^ conducted a systemic review of 31 randomized controlled trials (RCTs) (three in a clinical setting and 28 in a simulated setting). The newest RCTs (2004–2014) showed a clear benefit of 3D in comparison with 2D laparoscopy in almost 80% of trials as compared with only 40% of the older studies (1994–2004). This shows the positive impact of this technological advancement. In summary, the authors reported that 22 (71%) out of 31 trials showed a reduction in operation time and that 12 (63%) out of 19 showed a significant reduction in error when using 3D compared with 2D laparoscopy. In 2017, Patankar and Padasalagi performed a randomized study that compared conventional and 3D laparoscopy and that included 108 urological procedures, including 40 pyeloplasties ^35^. They reported a significant superiority of the 3D technique in terms of total operative time, dissection, suturing and stenting time, blood loss, and surgeon stress. At the same time, the hospital stay and complications were almost the same in these two groups. Similar results were presented by Tran *et al.* ^33^ after performing the first hundred cases of 3D laparoscopy, including 13 pyeloplasties. These results confirmed a significant reduction in operative time, blood loss, and time taken for critical steps such as dissection of ureteropelvic junction, creation of anastomotic flap, suturing, and stenting. A systematic review and meta-analysis of 13 studies, to assess the clinical and surgical efficacy of the 3D laparoscopic system in comparison with 2D laparoscopy, performed by Wang *et al.* ^36^ proved a superiority of 3D laparoscopic surgery for many urologic procedures such as partial nephrectomy or radical prostatectomy. However, these studies did not show a significant difference between the two systems in the pyeloplasty group. The authors concluded that 3D laparoscopic pyeloplasty could currently find a place between 2D laparoscopy and robotic surgery as it provides a good depth perception, comparable to that of a robotic system, at lower costs and easier handling. In addition, Kim *et al.* ^37^ did not prove the benefit of the robotic system in comparison with 3D laparoscopy in terms of suturing performance (a very important step in pyeloplasty) among well-trained laparoscopic surgeons. It also remains unclear whether the robotic system offers any advantages over 3D laparoscopy in terms of the learning curve ^38^.

## Stenting, no stenting, fibrin glue?

The first successful dismembered pyeloplasty from Anderson and Hynes was performed without ureteral stent. Anderson and Hynes commented on their technique: “We are convinced that the so called splinting of any anastomosis is not only unnecessary but it is against all the principles of plastic procedure, as it leads to infection and fibrosis at the line of suture and subsequent stricture. The line of anastomosis should be wide enough and so fashioned as to render any subsequent contraction innocuous” ^3^.

With the introduction of laparoscopic pyeloplasty, the placement of a double-J stent across the ureteropelvic junction became an indispensable step of the surgery as it facilitates performing of laparoscopic anastomosis and maintains ureteric caliber and anastomotic alignment. Furthermore, a stent is considered to lower the risk of urinoma formation, thereby reducing periureteric fibrosis and restenosis ^39^ and lower the impact of post-operative edema at the anastomotic site. A big disadvantage of stenting is that it can cause significant patient discomfort ^39, 40^. For example, Joshi *et al.* ^40^ claim that more than 80% of patients experienced stent-related pain affecting daily activities, 32% experienced sexual dysfunction, and 58% experienced reduced work capacity. For this reason, surgeons began to question the need for ureteral stenting for a successful laparoscopic pyeloplasty. Challenging the current practice to place the double-J stent for a period of 3 to 6 weeks, Danuser *et al.* ^41^ presented a prospective randomized single-center study with a total of 100 cases. They aimed at answering the question whether 1-week stenting of the ureteropelvic anastomosis of laparoscopic or robot-assisted pyeloplasty is as effective as the more traditional 4-week stenting. The authors proved that the success rate in the “1 week” series was not inferior to the success rate in the “4 week” series and there were no significant differences with regards to residual symptoms, rate of complications, improvement in split renal function, or duration of surgery. The significant advantage was a shorter length of hospital stay in the “1 week” series (5 versus 6 days). A further step was to investigate whether a ureteral stent is necessary at all. Since then, many authors have been able to prove that in both children and adults, regardless of whether classic laparoscopic or robot-assisted pyeloplasty is used, there is no significant difference between outcomes in stented and non-stented patients ^2, 6, 42– 45^. Although the risk of prolonged anastomotic leakage and hospital stay are initially higher in the non-stented pyeloplasty, success rates for stented and non-stented pyeloplasty are the same ^45^. The advantages of stentless pyeloplasty are the avoidance of stent-related morbidity and the need for a second intervention for stent removal which makes such an approach cost-effective ^2, 39^. Nevertheless, almost all authors emphasize that the stentless procedure should be performed only by an experienced surgeon and the decision should be made in light of patient characteristics and intra-operative findings ^44, 45^.

Some authors proposed, as an aid in performing a stentless procedure, that fibrin glue can be used as a sealant at the anastomotic line. Fibrin glue is a mixture of coagulation factors which can be used as a urinary tract sealant (mostly in urological anastomoses), tissue adhesive, and hemostatic agent ^39, 46^. In an RCT, Farouk *et al.* ^39^ proved that omitting stenting during laparoscopic pyeloplasty in combination with fibrin glue can significantly reduce the rate of early post-operative adverse events and complications. Outcomes were similarly favorable as in patients with classic stented laparoscopic pyeloplasty. Supporting a positive impact of using fibrin glue in the upper urinary tract, Wolf *et al.* ^47^ showed, in a porcine model, that laparoscopic closure of a ureterotomy with the use of fibrin glue displayed significantly higher flow rates than the control group and was superior in histological evidence of healing. Furthermore, Eden *et al.* ^48^ claimed that the use of fibrin glue can significantly shorten the operating time and hospitalization and lower the post-operative opiate analgesic requirement.

## Conclusions

A multitude of new technologies have been introduced in the treatment of ureteropelvic junction obstruction over the last three decades. However, the principle of the dismembered pyeloplasty as presented by Anderson and Hynes nearly 70 years ago is still valid. The concept of this open surgery technique has been successfully translated into the laparoscopic and later robotic approach. Recent technology has focused on improving the classic laparoscopic technique with, for example, 3D vision. To further minimize surgical trauma, a single-port access to the abdominal cavity has been developed. Another attempt to reduce patient post-operative discomfort was to omit a double-J stent. A fibrin glue has been successfully used instead of a double-J stent, improving post-operative outcomes. The introduction of the robotic system has simplified the most difficult part of the operation: the suturing. It is clear that minimally invasive surgery is the future for the treatment of ureteropelvic junction obstruction. Nevertheless, it is hard to conclude, on the basis of available data, which approach should be favored for pyeloplasty. Both the laparoscopic and the robotic approach are being refined and show excellent results. This is reassuring as—in the realities of a hospital—available instruments, surgeon experience, patient factors, and financial aspects play a key role and often dictate the surgeon’s choices.
